# Plant-Based Chocolate Desserts: Analysis of Consumer’s Response According to Sensory Properties of Products and Consumer Attitude Towards Meat Reduction

**DOI:** 10.1007/s11130-025-01338-3

**Published:** 2025-03-18

**Authors:** Franco D. Della Fontana, Gabriel López-Font, Djemaa Moussaoui, María C. Goldner, Carolina Chaya

**Affiliations:** 1 Department of Agricultural Economics, Statistics and Business Management, Universidad Politécnica de Madrid, Madrid, Spain; 2 Instituto de Investigaciones para la Industria Química, Universidad Nacional de Salta, Salta, Argentina; 3 Instituto de Investigaciones Sensoriales de Alimentos, Facultad de Ciencias de la Salud, Universidad Nacional de Salta, Salta, Argentina

**Keywords:** Brea Gum, Black Carob Flour, Soy, Acceptance, Emotional Response, Sustainable

## Abstract

Brea gum is a food additive with potential for economic and environmental sustainability. It may be used in plant-based formulations, such as chocolate desserts, but achieving acceptable sensory characteristics remains a challenge. Therefore, the aim was to compare the response of consumers to two plant-based chocolate desserts, under blind and informed conditions about ingredient formulation. Two plant-based chocolate desserts were used: one made with Brea gum and black carob flour (BG) and a commercial soy chocolate dessert (CS). Eight trained judges characterized the sensory properties of both products. 103 consumers (female = 62, aged between 18 and 45) tasted both products in blind and informed conditions and rated their overall liking, and their liking of appearance, odour, texture, sweetness, flavour, and vegetable aftertaste. Purchase intention and healthy, processed and sustainable perception were also rated. Participants also completed a questionnaire on attitudes towards reducing meat consumption. BG samples showed higher intensities of vegetable odour and vegetable aftertaste, while CS samples were significantly higher in sweetness and brown colour (*p* < 0.05). Acceptability was significantly higher in CS than in BG (7.1 ± 1.6 and 4.9 ± 2.2, respectively, *p* < 0.05). BG was perceived as more sustainable than CS (6.6 ± 2.3, 6.1 ± 2.2, respectively; *p* < 0.05). Information about the samples had no effect on overall liking. Supporters of meat reduction showed a higher acceptability for both desserts than rejecters. Acceptability of both plant-based desserts was influenced by intrinsic sensory characteristics and by consumers’ attitude towards meat reduction. Improvements in sensory attributes need further development.

**Supplementary Information:**

The online version contains supplementary material available at 10.1007/s11130-025-01338-3.

## Introduction

Globally, society is moving towards sustainable diets due to increased environmental and health concerns, as well as ethical considerations regarding animal welfare and more sustainable production systems [1, 2]. Sustainability involves what humans do with land/environment and how they do it to serve human society [1]. In this sense, South America’s indigenous communities have a deep-rooted history of plant-based eating, such as corn, Andean grains (quinoa, kiwicha, amaranto), pulses (such as black carob), fruits, and exudates (*e.g*., Brea gum) constituting the basis of their diets. For example, indigenous desserts often use these ingredients in traditional recipes, many of which are naturally plant-based.

Brea gum is a hydrocolloid obtained from the brea tree (*Parkinsonia praecox* or *Cercidium praecox*) that grows in arid or semi-arid regions of Argentina [2]. This gum is obtained sustainably by cutting into the trunk and branches of the brea tree. The collection process has been carried out by Wichí people since its origins. Wichí is one of several indigenous peoples that live in Salta, Chaco, and *Formosa* provinces in the north of Argentina [3], where children eat Brea Gum as a natural candy called ‘caramelo del Monte’. Although the chemical structures are different [4], Brea gum has been used as a substitute for Arabic gum (GA) due to its technological similarities tested on aqueous solutions [5], emulsions [6], foams [7], microencapsulation [8], hydrogels [9] and biofilms [10].

As a food additive, Brea gum has only been used in gluten-free bread, filled cookies, and pasta formulation [11–13]and has only been evaluated by Argentinian consumers with promising results, but no research was found with other populations. Since the demand for vegetable additives is growing worldwide [1], and cultural differences are reasons for product acceptance or rejection [14], it is important to study new developments in different countries. In Europe, there is a concern about promoting a more sustainable and healthy diet [15], which is why meat reduction consumption is expected [16].

According to Muñoz-Martinez et al. [15] Spanish consumers have become more aware of health and seek healthier alternatives in recent years. Traditional chocolate desserts could be replaced by plant-based products, including ingredients such as pulse flour and Brea gum, as suitable alternatives to enjoy something sweet that does not include animal products such as milk in their formulation. Additionally, among the novel plant-based products, desserts are an important market that reached USD 2.9 billion in 2022 and is projected to reach USD 58 billion by 2030 [17].

In this context of an increasing demand for plant-based products, understanding the preferences and opinions of consumers about plant-based foods such as chocolate desserts can provide valuable information for both the food industry and policymakers. Therefore, this study aimed to assess the effect of consumer attitudes toward the reduction of meat consumption on the consumer response of a semi-solid dessert with brea gum and black carob flour under blind and informed conditions.

## Materials and Methods

The Materials and Methods section is presented as supplementary material 1.

## Results and Discussion

### Sensory Evaluation with Trained Judges

The judges identified the differences between the high and low references for each attribute showing a good discrimination. There were no significant interactions between the references and the judges showing, a good internal coherence of the panel (Table S3). Significant interactions between the references and the sessions were found for the attributes of vegetable odour, consistency, sandy texture, and chocolate flavour. Although the references for these attributes were adequately assessed, a detailed evaluation of the interaction plots allowed the detection of unimportant differences in the measurements due to the use of the scales. In summary, the panel showed good discrimination between high and low references, good internal coherence among judges, and acceptable reproducibility between sessions for all the attributes during training.

After training, the judges conducted the sensory evaluation of the products in one session. The BG samples showed higher intensities of vegetable odour and vegetable aftertaste, while CS samples were significantly higher in sweetness and brown colour (Fig. S1) (*p* < 0.05).

### Consumer Study

#### Consumer Attitudes Towards Meat Reduction

The participants were mainly women (60%), mostly following a Mediterranean diet (89%), and 56% had a health or food-related background. Consumers completed a questionnaire on their attitude towards meat reduction according to different dimensions (Table S2). The mean scores for each dimension are shown in Table 1. A consumer classification was done according to low, medium, and high ratings of the questionnaire. The consumers were divided into three classes according to their attitude towards meat reduction: the first tertile was named Rejecters, the second tertile was named Intermediate and the third tertile was named Supporters. Hedonic dimension showed the lowest mean score in all groups, while health dimension had the highest in the Rejecters and Intermediate groups. Supporters group showed significantly higher scores in the environment, ethic and health dimensions. Hence, it is possible to identify the major dimensions influencing attitude towards meat reduction.

No significant differences were found according to health or food background. Significant differences were observed by gender in attitudes towards meat reduction. Women had significantly higher scores (3.82 ± 0.87) according to their attitudes towards meat reduction (t = 3.83, *p* < 0.001), meaning they were more supportive of meat reduction than men (3.11 ± 1.00). Other studies reported that women are more involved in food purchasing and preparation [18], which could lead to a higher awareness of food-related issues that men [19, 20].

**Table 1:** Mean scores and standard deviations of dimensions of attitude towards meat reduction according to consumer classes

Dimension	Consumer class					
	Rejecters (*n* = 33)		Intermediate (*n* = 35)		Supporters (*n* = 35)	
	*x̅*	SD	*x̅*	SD	*x̅*	SD
Hedonic	1.49a	0.6	2.19a	1.0	3.58a	1.2
Diet	3.10b	0.8	3.62bc	0.8	4.72bc	1.0
Habit	2.10c	0.8	3.15b	0.9	4.36b	1.0
Environment	3.13b	0.9	3.89c	1.0	5.24 cd	0.9
Ethic	2.17c	1.0	3.65bc	1.0	4.74bcd	1.2
Health	4.19d	0.9	4.95d	0.6	5.38d	0.8

Note: For each column, different letters indicate statistically significant differences between dimensions

#### Consumer Testing

Overall acceptability was significantly higher in CS than in BG (7.1 ± 1.6 and 4.9 ± 2.2, respectively). Significant differences in the hedonic response were observed for all sensory modalities and purchase intention, whether the test was performed under blind or informed conditions (Table 2). The only exception was the odour acceptability, which showed non-significant differences between samples under blind conditions. BG desserts were close to a neutral hedonic rating from the consumers, according to mean scores of overall acceptability; this suggests that although certain aspects of its sensory quality need to be improved, Brea gum may still be a good choice for this type of product. Non-significant differences were found on healthy and processed perception, but BG was perceived to be more sustainable than CS (6.6 ± 2.3 vs. 6.1 ± 2.2, respectively). In summary, the information did not affect the consumer’s hedonic response, purchase intention, and healthy, processed, and sustainable perception.

**Table 2:** Means and SD for consumer liking, purchase intention and healthy/processed/sustainable perception under blind and informed conditions

Attribute	Blind Conditions					Informed conditions				
	BG		CS		*p*	BG		CS		*p*
	*x̅*	SD	*x̅*	SD		*x̅*	SD	*x̅*	SD	
Overall liking	4.79	2.2	7.13	1.6	< 0.001	4.61	2.3	6.95	2.0	< 0.001
Appearance liking	7.11	1.7	7.51	1.3	0.024	6.55	1.7	7.40	1.3	< 0.001
Odour liking	5.85	1.7	6.25	1.7	0.061	5.62	1.9	6.40	1.6	< 0.001
Texture liking	5.28	2.1	7.46	1.4	< 0.001	5.22	2.2	7.34	1.4	< 0.001
Sweetness liking	4.76	2.2	6.98	1.5	< 0.001	4.63	2.2	7.09	1.7	< 0.001
Flavour liking	4.50	2.2	7.04	1.7	< 0.001	4.26	2.4	6.97	2.1	< 0.001
Aftertaste liking	4.22	2.1	6.46	1.8	< 0.001	4.23	2.1	6.45	2.1	< 0.001
Purchase intention	2.26	1.1	3.62	1.0	< 0.001	2.31	1.2	3.78	1.2	< 0.001
Healthy perception	-	-	-	-	-	6.28	2.2	5.99	2.2	0.163
Processed perception	-	-	-	-	-	4.92	2.7	5.13	2.4	0.379
Sustainable perception	-	-	-	-	-	6.64	2.7	6.08	2.2	0.008

The BG dessert had lower liking ratings that confirmed that sensory quality was the main driver of consumer acceptance. On the contrary, the commercial dessert had significantly higher ratings on acceptability and purchase intention. Additionally, the sensory description of the trained judges was useful to identify sweetness and typical chocolate brownness as sensory drivers of liking in the commercial sample. This result was in line with De Medeiros et al. [21, 22] who found that plant-based chocolate desserts with soy protein were the most preferred due to the low intensity of chocolate flavour and the high sweetness. Cardello et al. [23] reported similar drivers of liking for plant-based yoghurts. Moreover, Falkeisen et al. [24] and Moss et al. [25] found that the main driver of disliking plant-based cheese and milk alternatives were beany and vegetable off flavour, which was in line with the drivers of disliking found in this study (vegetable odour and vegetable aftertaste). These findings show that the development of plant-based products using Brea gum with adequate sensory acceptance remains challenging and needs further improvements. Furthermore, since BG dessert was not available on the Spanish market, and familiarity plays a significant role in consumer expectations [26]- especially as one of the biggest barriers to meat reduction [27] - the lack of familiarity with BG dessert could be another reason for its low acceptability rating.

Liking and purchase intention were not affected by the information provided (*p* > 0.05) (Table S4). Therefore, as other studies found [28], sensory profiling was the main driver because the composition information of the desserts barely influenced sensory perception. During the informed evaluation, a statement about the benefits of Brea gum for the environment was presented, which might partially have contributed to the healthy, processed and sustainable perception. Jaeger et al. [29] found a limited effect of health or environmental information on the consumer response of plant-based yoghurts. In this sense, the latter authors suggest that the information should be more persuasive and not simply factual in order to develop effective strategies in the promotion of plant-based food.

#### Emotional Response

The CS dessert had a major elicitation of positive emotions compared to the BG desserts. Having information on the composition of the BG sample evoked significantly higher citations of good and free feelings and significantly lower citations of aggressive feelings than CS (Table S3). This finding was consistent with other studies [28, 29] that postulate that emotions are mainly sensory driven, and that information alone has a limited impact on emotional profile. Furthermore, Falkeisen et al. [24]and Moss et al. [25] found that alternatives to cheese and milk were considered healthy and sustainable, with high overall liking scores associated with positive emotions. They found that the emotional responses seem to agree with the results of the hedonic scales for different plant-based alternatives, as the most liked products were associated with positive emotions and the least liked with negative emotions. In the same line, Jaeger et al. [30] studied the sensory drivers of liking and emotional, holistic and conceptual associations from plant-based yoghurts. They found that words with positive meanings were associated with hedonic lifts, while those with negative meanings were associated with hedonic penalties. According to our results, this was probably due to the disparity between the sensory expectations of consumers, mainly based on their experience with regular chocolate desserts, the lack of familiarity with the BG dessert, and the actual experience when tasting the plant-based formulations.

### Influence of Attitudes Towards Meat Reduction on Consumer Response

Comparisons of hedonic response and purchase intention were made according to the classification of consumers by their attitudes towards meat consumption reduction. Therefore, supporters and intermediate showed significantly higher overall liking and purchase intention ratings than rejecters for both samples under blind conditions (*p* < 0.05) (Table 3 and Table S6). In particular, vegetable aftertaste ratings under blind conditions had a significant interaction in both consumer classes and type of sample (*p* < 0.05) showing that rejecters rated the BG sample with lower scores compared to CS. When information was provided, a significant effect of the consumer class was also observed for overall liking and purchase intention. Furthermore, under informed conditions, the consumer class had a significant effect on the degree of liking of texture, sweetness, and flavour showing higher ratings in supporters and intermediates than in rejecters (*p* < 0.01). Relevant differences in liking and purchase intention between consumer classes were observed for BG samples.

**Table 3:** Hedonic response and purchase intention of chocolate desserts according to consumers’ attitude towards meat reduction

Blind condition												
Attribute	BG						CS					
	Rejecters		Intermediate		Supporters		Rejecters		Intermediate		Supporters	
	*x̅*	SD	*x̅*	SD	*x̅*	SD	*x̅*	SD	*x̅*	SD	*x̅*	SD
Overall liking	3.97a	2.2	5.21b	2.1	5.24b	2.1	6.97a	1.8	7.09a	1.4	7.33a	1.5
Appearance liking	6.69a	2.0	7.18a	1.4	7.48a	1.5	7.36a	1.3	7.50a	1.1	7.70a	1.6
Odour liking	5.64a	1.8	5.82a	1.7	6.12a	1.7	6.56a	1.5	5.91a	1.8	6.27a	1.6
Texture liking	4.97a	2.2	5.53a	1.9	5.36a	2.2	7.33a	1.4	7.18a	1.6	7.88a	1.2
Sweetness liking	4.19a	2.4	4.85a	2.0	5.27a	2.1	7.03a	1.5	6.91a	1.5	7.00a	1.5
Flavour liking	3.72a	2.1	4.88a	2.1	4.94a	2.3	6.92a	1.9	6.97a	1.6	7.24a	1.5
Aftertaste liking	3.19a	2.1	4.68b	1.9	4.88b	1.9	6.47a	1.8	6.26a	1.7	6.64a	1.9
Purchase intention	1.83a	1.0	2.38ab	1.1	2.61b	1.1	3.47a	1.2	3.59a	1.0	3.82a	1.0

Informed condition												
Attribute liking	BG						CS					
	Rejecters		Intermediate		Supporters		Rejecters		Intermediate		Supporters	
	*x̅*	SD	*x̅*	SD	*x̅*	SD	*x̅*	SD	*x̅*	SD	*x̅*	SD
Overall liking	3.81a	2.5	4.88ab	2.0	5.21b	2.0	6.28a	2.3	7.26a	1.4	7.36a	1.9
Appearance liking	6.11a	1.8	6.74a	1.6	6.85a	1.5	7.14a	1.4	7.50a	1.0	7.58a	1.5
Odour liking	5.25a	2.2	5.65a	1.7	6.00a	1.7	6.19a	1.6	6.47a	1.4	6.55a	1.8
Texture liking	4.31a	2.4	5.50ab	2.0	5.94b	1.9	6.94a	1.6	7.26ab	1.1	7.85b	1.1
Sweetness liking	3.72a	2.4	5.00b	1.8	5.24b	1.9	6.61a	2.1	7.29a	1.2	7.39a	1.5
Flavour liking	3.25a	2.6	4.88b	2.1	4.73b	2.1	6.36a	2.4	7.21a	1.6	7.39a	2.1
Aftertaste liking	3.28a	2.2	4.91b	1.8	4.58b	2.0	6.03a	2.3	6.85a	1.4	6.48a	2.3
Purchase intention	1.83a	1.1	2.56b	1.1	2.58b	1.1	3.47a	1.3	3.88a	0.9	4.00a	1.3

Note: For each row and sample, different letters indicate statistically significant differences between consumer classes

Not surprisingly, the hedonic response and purchase intention were affected by attitudes toward meat reduction, showing higher liking ratings for supporters than for rejecters. Similarly, Moussaoui et al. [31] found that the expected liking of plant-based burgers was significantly higher among supporters than in rejecters. In this sense, Moussaoui et al. [31] and Graça et al. [32] concluded that the attitude towards meat reduction improved consumers’ preferences for plant-based products. Furthermore, Pointke et al. [33] found that overall liking for three different plant-based alternatives was significantly higher in vegans than in omnivores. The authors stated that omnivores and flexitarians, who regularly consume animal products, have a different sensory expectation and response to plant-based products than vegetarians and vegans [33]. Regarding purchase intention, Denver et al. [34] found that improving taste, expectations and reducing price are ways to reduce barriers to the consumption of plant-based products. Our research did not scope calculation of costs and sales prices which could affect supporters and rejecters’ attitudes, and therefore emphasise purchase intention ratings.

The comparison of healthy, processed, and sustainable perception was also made according to the classification of consumers by their attitude toward meat reduction. There was no significant effect of the classes of consumer on healthy or processed perception. However, supporters of meat reduction considered the BG sample to be more sustainable than rejecters (*p* < 0.05). Cliceri et al. [35] attribute these differences to positive attitudes towards plant-based dishes, which were positively related to empathic sensitivity towards humans and animals. In addition, these authors highlighted an important role for food awareness in determining eating habits and attitudes towards healthy and natural products. Furthermore, sustainability is a determining factor that motivates consumers to reduce their meat consumption and prefer plant-based products [16]. This concern for the environment might explain their greater inclination towards this type of product. Therefore, it is pertinent to suggest that consumers who follow plant-based food patterns are of particular significance for future food product development.

## Conclusion

The acceptability of plant-based desserts was strongly influenced by sensory characteristics and consumer attitudes towards meat reduction, independently of the information provided. The main drivers of liking for this type of product were sweetness and colour according to the description of trained judges. The hedonic response and purchase intention were also affected by attitudes towards meat reduction, showing higher liking ratings for supporters than for rejecters. Supporters of meat reduction considered desserts with Brea gum to be more sustainable than rejecters.

These results show that consumers’ attitude towards meat consumption reduction plays an important role in perception of plant-based products, and these variations can be valuable to the industry when developing products tailored to the preferences of each segment of consumers. Improvements in sensory attributes should also be considered, such as the addition of other ingredients to mask vegetable odour and aftertaste. In addition, informational strategies are needed to better understand the potential of sustainable plant-based desserts. Furthermore, we believe that more studies on the utilisation of Brea gum contribute to the preservation of biodiversity and the enhancement of resilience in agricultural systems.

## Electronic Supplementary Material

Below is the link to the electronic supplementary material.

Supplementary Material 1

Supplementary Material 2

## Data Availability

No datasets were generated or analysed during the current study.
