# Supplementary material for: Plant-Based Chocolate Desserts: Analysis of Consumer’s Response According to Sensory Properties of Products and Consumer Attitude Towards Meat Reduction
Source: Plant Foods Hum Nutr. 2025 Mar 18;80(2):93. doi: 10.1007/s11130-025-01338-3 (PMC11920308; doi:10.1007/s11130-025-01338-3)
Supplement: Supplementary file 1 — Supplementary Material 1 [file 11130_2025_1338_MOESM1_ESM.pdf]

Plant-based chocolate desserts: analysis of consumer's response according to sensory properties of products and consumer attitude towards meat reduction

Franco D. Della Fontana[1,2,3], Gabriel López-Font[1], Djemaa Moussaoui[1], María C. Goldner[2,3], Carolina Chaya[1]

[1] Department of Agricultural Economics, Statistics and Business Management, Universidad Politécnica de Madrid, Madrid, Spain

[2] Instituto de Investigaciones para la Industria Química, Universidad Nacional de Salta, Salta, Argentina

[3] Instituto de Investigaciones Sensoriales de Alimentos, Facultad de Ciencias de la Salud, Universidad Nacional de Salta, Salta, Argentina

Corresponding author: Carolina Chaya. ORCID: https://orcid.org/0000-0002-5518-886X E-mail address: carolina.chaya@upm.es

Material and methods

Sample preparation

Preliminary tastings with the research team were conducted in order to achieve an acceptable product for this study, from the sensory point of view (data not shown). The selected chocolate Brea gum dessert (BG) was prepared using the following proportions of ingredients (for 100 ml): black carob flour 5%, defatted cocoa powder 5%, maize starch 5%, sucrose 10% and brea gum 1%. Black carob flour, cocoa powder, sucrose, and maize starch were purchased in local markets. Brea gum was provided by indigenous communities of Chaco Salteño (Argentina).

The methods described by Seuvré [1] and Aguilar-Raymundo [2] were adapted for sample preparation. First, all dry ingredients were weighed and mixed in a flask. Then, water was added to the corresponding volume and stirred at 1000 rpm in a magnetic

stirrer for 10 minutes. Subsequently, the mixture was heated using an electric plate (Barnstead/Thermolyne, Dubuque, IA, USA) to 90 ° C. After heating, the desserts were cooled at room temperature and stored at 4 ° C for 24 hours. Commercial samples of plant-based chocolate desserts were purchased at local markets in Madrid. The selected commercial product was a chocolate dessert made with soy drink (CS).

Sensory evaluation with trained judges

Eight volunteer assessors with previous sensory evaluation experience were trained in the descriptive analysis of chocolate desserts. Training was done in the recognition of the descriptors associated with the chocolate desserts, based on the provision of suitable references for each descriptor. Once a consensus was reached on the terms by the assessor panel, two references for each descriptor were prepared at low and high concentrations (Table S1). Reference samples were coded using 3-digit random numbers and the order of presentation was balanced. Ballots with unstructured 10-cm line scales for each descriptor that were anchored from the ends of both extremes with the labels 'low' and 'high', respectively, were presented to the assessors. Special attention was paid to the statistical analysis of assessor discrimination, internal coherence, and reproducibility. When the entire panel was able to discriminate all attributes and correctly rate their intensity, the sensory evaluation of both desserts was performed. Training was carried out in two sessions of two hours, while sensory evaluation of the products was carried out in one session of two hours.

For the chocolate dessert evaluation, 30 g of both products (BG and CS) were served in 30 ml paper containers wrapped with aluminium paper to prevent volatile loss. Samples were coded using 3-digit random numbers and the order of presentation was balanced. The assessors were instructed to rate the intensity of each attribute on an unstructured 10-cm line scale. The panelists performed the evaluation in blind conditions, i.e. they

were not informed about the nature of the samples. The panelists were asked not to eat or drink anything two hours before the tests. Low-mineral water was administered to rinse their palates between sample tastings. The training sessions were conducted by members of the SensUPM team and all tests were carried out in the SensUPM sensory laboratory (Department of Agricutural Economics, Statistics, and Business Management, Madrid, Spain). All training and sensory evaluation data were collected on tablets using Compusense Cloud Software (Compusense, Inc. 2023, Guelph, Ontario, Canada).

Consumer study

One hundred and three consumers (women = 62) between 18 and 45 years old (23 ± 5.7) participated in this study. Volunteers were invited to participate through university advertisements (Universidad Politécnica de Madrid) and emails sent to the consumer panel (SensUPM). Participants were organised into different sessions to have a limited number of respondents in each specific session (between 10 and 15). Once at the university facilities, the data of the participants was collected using computers. The questionnaire was administered using Compusense Cloud Software (Compusense, Inc. 2023. Guelph, Ontario, Canada).

Chocolate dessert samples (CS and BG) were served in sustainable paper cups. The products were coded using 3-digit random numbers. The order of presentation was balanced. Each consumer evaluated both products under blind and informed conditions, in one session. First, participants were asked to rate their liking on a 9-point hedonic scale (labelled from "dislike extremely" to "like extremely") and purchase intention on a 5-point scale (from "Definitely I wouldn't buy it" to "Definitely I would buy it") under blind conditions (without ingredient information). Second, consumers were asked to choose the emotions elicited by the samples with a validated version of the EsSense25

lexicon in Spanish [3, 4]. The emotions were randomised for each consumer, but the same order was always kept for each consumer across the different samples.

Table S1. Definitions of attributes and references selected for sensory evaluation with trained panelists

Attribute	Definition	References	
		Low	High
Consistency	Resistance to force made by a plastic spoon	Plant-based chocolate dessert with 2.5% starch	Plant-based chocolate dessert with 10% starch
Brownness	Brown colour characteristic of products made with chocolate	Commercial chocolate dessert	Plant-based chocolate dessert with 10% cocoa
Chocolate odour	Characteristic aroma of cocoa powder	Plant-based chocolate dessert with 1% cocoa	Plant-based chocolate dessert with 10% cocoa
Vegetable odour	Aroma characteristic of vegetables	Plant-based chocolate dessert with 0.5% pea protein isolate	Plant-based chocolate dessert with 5% pea protein isolate
Sweetness	Sweet taste characteristic of adding sucrose or sweetener.	Plant-based chocolate dessert with 2.5% sucrose	Commercial chocolate dessert
Chocolate flavour	Characteristic cocoa flavour.	Plant-based chocolate dessert with 1% cocoa	Plant-based chocolate dessert with 10% cocoa
Firmness	Force required to deform the product by compressing it between the tongue and the palate.	Plant-based chocolate dessert with 2.5% starch	Plant-based chocolate dessert with 10% starch
Sandy texture	Perception of small particles on the tongue.	Plant-based chocolate dessert with 0.5% pea protein isolate	Plant-based chocolate dessert with 5% pea protein isolate
Vegetable aftertaste	Vegetable flavour after swallowing the product.	Plant-based chocolate dessert with 0.5% pea protein isolate	Plant-based chocolate dessert with 5% pea protein isolate

After collecting data on the blind condition, consumers were asked to rest for 30 seconds and rinse their mouths with water. Then, they were asked to repeat the hedonic and emotional evaluation on the same samples, but this time they received information

about the dessert ingredients. For the CS dessert, the information provided was '*Commercial and vegan chocolate dessert made with soy*' and for the BG dessert it was '*Vegan chocolate dessert made with carob flour and Brea gum. Brea gum is an ingredient extracted from a native Argentine tree with minimal impact on its vegetative development. Its manual extraction contributes to regional employment and economy'*. Furthermore, in informed condition, consumers were asked to rate the healthy, processed, and sustainable perception of the samples on 10-cm line scales. The scales were anchored on the extremes with labels 'not healthy' to 'very healthy', 'natural / not processed' to 'very processed' and 'not sustainable' to 'very sustainable', respectively.

The final part of the questionnaire included questions about consumer attitudes towards meat reduction, based on previous studies [5, 6]; the complete description of the items can be found in Moussaoui et al. [5]. Participants were asked to indicate their degree of agreement with 32 statements (motivated to diet, habits, ethics, hedonism, health, and the environment) (Table S2) using a 7-point Likert scale (from 'strongly disagree' to 'strongly agree'). Thirteen items were reversed and their scores were processed properly prior to data analysis [6]. The reversal of some items is intended to keep consumers' attention and to minimise the halo effect. The items in the questionnaire were randomised to avoid bias. Data regarding participant´s diet and health or food-related background were also collected.

Consumers were acknowledged with chocolates and/or sweets at the end of the session. The study was carried out in accordance with the principles laid down in the Declaration of Helsinki, and all subjects signed a written informed consent at the beginning of the test. The Ethics Committee of the Universidad Nacional de Salta (Res-CD-212/2020) approved the study protocols.

Data analysis

Concerning the statistical evaluation of training, for each attribute, two-way ANOVAs on attribute intensities were performed to check discrimination between samples, coherence among panelists, and reproducibility between the two training sessions of the panel sensory assessments. To this end, reference and panelist were used as fixed factors in the first ANOVA, and reference and session were used as fixed factors in the second ANOVA. Once the training was completed, the data provided by the sensory panel of the two products were analysed by two-way ANOVAs for each attribute, with the sample and the panelist as fixed effects [7]. t-tests were performed on the intensity of each attribute to analyse the differences between the two samples.

Concerning consumer tests, a mean score for each participant was calculated from the 32 statements on the attitudes towards meat reduction questionnaire. Then, the tertiles of the mean scores were calculated to form three consumer groups: Supporters (consumers who were willing to reduce their meat consumption, mean scores higher than the second tertile), Rejecters (consumers who were not willing to reduce their meat consumption, mean scores below the first tertile), and Intermediate (an intermediate attitude, mean scores between the first and second tertiles). t-tests were performed to identify differences in mean scores by gender and by health or food-related background, and one-way ANOVAs were performed to assess differences between consumer diets. Normality was checked with Shapiro-Wilks test, and nonparametric tests were performed when normality was not verified.

Two-way ANOVAs were performed to assess the effect on liking, purchase intention, healthy, processed, and sustainable perception, according to the following fixed factors: sample (BG/CS), test condition (blind/informed) and consumer class (rejecters/ intermediate/ supporters). When appropriate, two-way interactions were considered

(sample × consumers' class). When applicable, the consumer was also included in the models as a random effect. Fisher LSD tests were used for ANOVA significant factors to test significant differences between means.

Table S2. Statements used for the measurement of the attitude towards meat reduction.

Dimension	Statement	Reversed
Diet 1	I need to eat meat to have enough energy.	Yes
Diet 2	All things considered. meat is necessary in the human.	Yes
Diet 3	If I couldn't eat meat, I would feel weak.	Yes
Diet 4	Diet 4 Eating meat is important for a complete diet.	Yes
Diet 5	Eating meat is part of a balanced lifestyle.	No
Diet 6	It is possible to have an adequate diet without eating meat.	No
Diet 7	Meat is irreplaceable in my diet.	Yes
Environment 1	Eating meat has a negative impact on the environment.	No
Environment 2	By eating meat, I support an industry which is responsible for environmental damage.	No
Environment 3	By eating meat. I'm also responsible for the problems associated with its production.	No
Environment 4	To eat meat is disrespectful towards life and the environment.	No
Ethic 1	It would be difficult for me to watch an animal being killed for food purposes.	No
Ethic 2	If I had to kill the animals myself. I would probably stop eating meat.	No
Ethic 3	If I saw an animal being killed. I would have no problems eating it.	Yes
Ethic 4	I feel bad when I think about eating meat because of the animal suffering.	No
Ethic 5	Eating meat reminds me of the death and suffering of the animals.	No
Ethic 6	When I think about eating meat, I feel guilty.	No
Habit 1	I don't picture myself without eating meat regularly.	Yes
Habit 2	I would feel fine with a meatless diet.	No
Habit 3	It is easy to have a meat-free diet.	No
Health 1	Eating meat frequently is not bad for your health.	Yes
Health 2	Eating meat in excess has a negative impact on health.	No
Health 3	A diet with lots of meat can be harmful to health.	No
Health 4	If I ate less meat. my health would improve.	No
Health 5	Eating less meat is good for my health.	No
Hedonic 1	I love eating meat very much.	Yes
Hedonic 2	I love meals with meat.	Yes
Hedonic 3	I am a big fan of meat.	Yes
Hedonic 4	Eating meat is one of the good pleasures in life.	Yes
Hedonic 5	Meat disgusts me.	No
Hedonic 6	Nothing can compare with a good steak.	Yes
Hedonic 7	I do not like the taste of meat.	No

Source: Extracted from Moussaoui et al. [5]

The nonparametric Cochran Q test was applied to compare the emotions frequencies elicited for both samples under blind and informed test conditions, and the post hoc McNemar test was performed using multiple pairwise comparisons.

Different statistical analyses were performed at the respective stages of the study. When appropriate, a significance level of 0.05 was used. InfoStat (v2020) [8] and Statgraphics Centurion XIX (Statgraphics Technologies, Inc 2022, Virginia, United States) were used as statistical software.

References

1. Seuvre A-M, Turci C, Voilley A (2008) Effect of the temperature on the release of aroma compounds and on the rheological behaviour of model dairy custard. Food Chemistry 108:1176–1182. https://doi.org/10.1016/j.foodchem.2007.04.031
2. Aguilar-Raymundo VG, Vélez-Ruiz JF (2018) Physicochemical and Rheological Properties of a Dairy Dessert, Enriched with Chickpea Flour. Foods 7:25. https://doi.org/10.3390/foods7020025
3. Dorado R, Pérez-Hugalde C, Picard A, Chaya C (2016) Influence of first position effect on emotional response. Food Quality and Preference 49:189–196. https://doi.org/10.1016/j.foodqual.2015.12.009
4. Nestrud MA, Meiselman HL, King SC, et al (2016) Development of EsSense25, a shorter version of the EsSense Profile®. Food Quality and Preference 48:107–117. https://doi.org/10.1016/j.foodqual.2015.08.005
5. Moussaoui D, Torres-Moreno M, Tárrega A, et al (2023) Evaluation of consumers' response to plant-based burgers according to their attitude towards meat reduction. Food Quality and Preference 110:104955. https://doi.org/10.1016/j.foodqual.2023.104955

6. Tarrega A, Rizo A, Murciano A, et al (2020) Are mixed meat and vegetable protein products good alternatives for reducing meat consumption? A case study with burgers. Current Research in Food Science 3:30–40. https://doi.org/10.1016/j.crfs.2020.02.003
7. Chaya Romero C, Pérez-Hugalde C (2022) Métodos estadísticos para el análisis de datos sensoriales. In: Análisis sensorial de alimentos y respuesta del consumidor, 1st ed. Acribia, Zaragoza
8. Di Rienzo J, Casanoves F, Balzarini M, et al (2020) InfoStat
