# Supplementary material for: Plant-Based Chocolate Desserts: Analysis of Consumer’s Response According to Sensory Properties of Products and Consumer Attitude Towards Meat Reduction
Source: Plant Foods Hum Nutr. 2025 Mar 18;80(2):93. doi: 10.1007/s11130-025-01338-3 (PMC11920308; doi:10.1007/s11130-025-01338-3)
Supplement: Supplementary file 2 — Supplementary Material 2 [file 11130_2025_1338_MOESM2_ESM.pdf]

Plant-based chocolate desserts: analysis of consumer's response according to sensory properties of products and consumer attitude towards meat reduction

Franco D. Della Fontana[1,2,3], Gabriel López-Font[1], Djemaa Moussaoui[1], María C. Goldner[2,3], Carolina Chaya[1]

[1] Department of Agricultural Economics, Statistics and Business Management, Universidad Politécnica de Madrid, Madrid, Spain

[2] Instituto de Investigaciones para la Industria Química, Universidad Nacional de Salta, Salta, Argentina

[3] Instituto de Investigaciones Sensoriales de Alimentos, Facultad de Ciencias de la Salud, Universidad Nacional de Salta, Salta, Argentina

Corresponding author: Carolina Chaya. ORCID: https://orcid.org/0000-0002-5518-886X E-mail address: carolina.chaya@upm.es

Supplementary results

Table S3. Results of the statistical analysis of the training sessions.

Attribute	p-values		
	Reference	Reference*panelist	Reference*Session
**Brownness**	<0.0001	0.6609	0.1882
**Chocolate odour**	<0.0001	0.2940	0.3715
**Vegetable odour**	<0.0001	0.1375	0.0001
**Consistency**	<0.0001	0.7538	0.0025
**Firmness**	<0.0001	0.1960	0.6338
**Sandy texture**	<0.0001	0.4984	0.0185
**Sweetness**	<0.0001	0.2160	0.4859
**Chocolate flavour**	<0.0001	0.8034	0.0292
**Vegetable aftertaste**	<0.0001	0.0693	0.3686

Fig. S1 Samples description according to trained panel evaluation

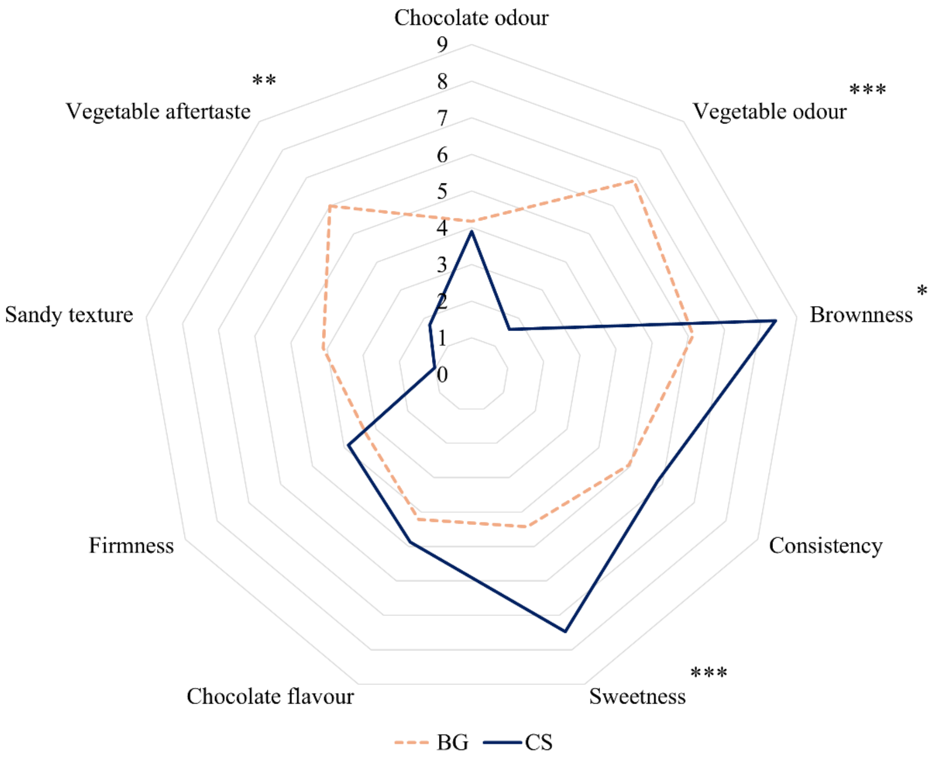

BG: brea gum plant-based chocolate dessert, CS: commercial soy plant-based chocolate dessert

*p<0.1, **p<0.05, ***p<0.001 for sample effect in two-way ANOVAs.

Table S4. p-values of the 2-way ANOVAs on consumer according to sample (BG/CS) and test condition (blind/informed)*

Attribute	Sample	Test condition	Interaction
**Overall liking**	**<0.001**	0.3769	>0.9999
**Appearance liking**	**<0.001**	**0.0247**	0.1423
**Odour liking**	**<0.001**	0.7980	0.2678
**Texture liking**	**<0.001**	0.6263	0.8711
**Sweetness liking**	**<0.001**	0.9590	0.5371
**Flavour liking**	**<0.001**	0.4693	0.6915
**Aftertaste liking**	**<0.001**	>0.9999	0.9612
**Purchase intention**	**<0.001**	0.3591	0.6308

*Bold values indicate statistically significant differences (p<0.05).

Table S5. Frequency counts of EsSense25 emotions elicited for each sample according to test conditions

Emotion	BG		p-value*	CS		p-value
	Blind	Informed		Blind	Informed	
Enthusiastic	1	2	0.564	13	8	0.251
Happy	13	7	0.083	34	34	1.000
Good natured	4	3	0.655	1	3	0.317
Free	1	5	**0.046**	7	9	0.564
Joyful	6	11	0.132	26	30	0.465
Interested	14	20	0.180	21	21	1.000
Understanding	2	3	0.564	1	3	0.157
Pleasant	19	12	0.127	40	37	0.639
Good	10	18	**0.046**	28	24	0.465
Adventurous	4	6	0.317	7	4	0.366
Secure	3	6	0.257	17	15	0.593
Active	6	5	0.739	13	7	0.058
Satisfied	19	15	0.371	49	44	0.353
Loving	1	1	1.000	4	5	0.655
Warm	2	1	0.564	3	9	0.058
Calm	19	11	0.074	23	23	1.000
Aggressive	5	1	**0.046**	0	1	0.317
Nostalgic	5	4	0.705	4	2	0.317
Wild	3	4	0.564	1	4	0.180
Tame	25	21	0.394	6	3	0.317
Mild	20	24	0.450	28	36	0.170
Guilty	3	4	0.655	0	0	1.000
Worried	13	10	0.439	2	2	1.000
Bored	19	11	0.074	6	6	1.000
Disgusted	26	35	0.061	4	7	0.317

*Bold values indicate statistically significant differences ($p<0.05$) according to Cochrane's Q test and McNemar post hoc comparisons. BG: brea gum plant-based chocolate dessert, CS: commercial soy plant-based chocolate dessert

Table S6. p-values of the 2-way ANOVAs on consumer response according to sample (BG/CS) and consumer class (rejecters/intermediate/supporters).

	**Blind**			**Informed**		
	**Sample**	**Consumer class**	**Sample * Consumer Class**	**Sample**	**Consumer class**	**Sample * Consumer Class**
Overall liking	**<0.0001**	**0.0230**	0.1685	**<0.0001**	**0.0008**	0.8958
Appearance liking	0.0575	0.0907	0.6493	**0.0001**	**0.0467**	0.8107
Odour liking	0.1058	0.5158	0.2772	**0.0020**	0.1816	0.7950
Texture liking	**<0.0001**	0.3177	0.3330	**<0.0001**	**0.0002**	0.2868
Sweetness liking	**<0.0001**	0.2621	0.2068	**<0.0001**	**0.0006**	0.4715
Flavour liking	**<0.0001**	0.0507	0.2070	**<0.0001**	**0.0006**	0.5595
Aftertaste liking	**<0.0001**	**0.0140**	**0.0154**	**<0.0001**	**0.0014**	0.3861
Purchase intention	**<0.0001**	**0.0082**	0.3806	**0.0019**	**<0.0001**	0.7083

*Bold values indicate statistically significant differences (p<0.05).
